# Feasibility of a randomized controlled trial to assess treatment of Middle East Respiratory Syndrome Coronavirus (MERS-CoV) infection in Saudi Arabia: a survey of physicians

**DOI:** 10.1186/s12871-016-0198-x

**Published:** 2016-07-12

**Authors:** Yaseen M. Arabi, Farhan Al-Enezi, Kajsa-Stina Longuere, Hanan H. Balkhy, Mohamed Al-Sultan, Awad Al-Omari, Fahad M. Al-Hameed, Gail Carson, Nahoko Shindo, Robert Fowler

**Affiliations:** 1 Intensive Care Department, King Saud bin Abdulaziz University for Health Sciences, King Abdullah International Medical Research Center, PO Box 22490, Riyadh, 11426 Saudi Arabia; 2 University of Oxford Centre for Tropical Medicine (CCVTM), Churchill Hospital Old Road, Headington, Oxford, OX3 7LE UK; 3 Infection Control Department, King Saud bin Abdulaziz University for Health Sciences, King Abdullah International Medical Research Center, Riyadh, Saudi Arabia; 4 Emergency & Critical Care Medicine, King Saud bin Abdulaziz University for Health Sciences, King Abdullah International Medical Research Center, Riyadh, Saudi Arabia; 5 AlFaisal University, Riyadh, Saudi Arabia; 6 Critical Care & Pulmonary Medicine, King Saud bin Abdulaziz University for Health Sciences, King Abdulaziz Medical City, Jeddah, Saudi Arabia; 7 Pandemic and Epidemic Diseases Department, World Health Organization, Geneva, Switzerland; 8 Department of Medicine and Institute of Health Policy Management and Evaluation, University of Toronto, Toronto, Canada; 9 Department of Medicine and Interdepartmental Division of Critical Care Medicine, Sunnybrook Health Sciences Centre, Toronto, Canada; 10 International Severe Acute Respiratory & emerging Infection Consortium (ISARIC), Old Road Campus, Headington, OX3 7FZ UK

**Keywords:** Middle East respiratory syndrome coronavirus (MERS-CoV), Convalescent plasma, Randomized controlled trial

## Abstract

**Background:**

The Middle East Respiratory Syndrome coronavirus (MERS-CoV) is an emerging respiratory pathogen with a high mortality rate and no specific treatments available to date. The purpose of this study was to determine the feasibility of conducting a randomized controlled trial (RCT) of convalescent plasma therapy for MERS-CoV-infected patients by using MERS-CoV-specific convalescent plasma obtained from previously recovered patients.

**Methods:**

A survey was adapted from validated questionnaire originally aimed to measure network capacities and capabilities within the International Severe Acute Respiratory and emerging Infection Consortium (ISARIC). The questionnaire was modified for this study to include 26 items that were divided into three main domains of interest: (1) the ability to care for critically ill MERS-CoV patients; (2) laboratory capacity to diagnose MERS-CoV and blood bank ability to prepare convalescent plasma; and (3), research capacity to conduct randomized controlled trials. The questionnaire was emailed to physicians.

**Results:**

Of 582 physicians who were invited to the survey, 327 responded (56.2 %). The professional focus of the majority of respondents was critical care (106/249 (43 %)), pediatrics (59/249, (24 %)) or internal medicine (52/249 (21 %)) but none was blood banking. Nearly all respondents (251/263 (95 %)) reported to have access to ICU facilities within their institutions. Most respondents (219/270 (81 %)) reported that intensivists were the most physician group responsible for treatment decisions about critically ill SARI patients. While 125/165 respondents (76 %) reported that they conduct research in ICUs, and 80/161 (49.7 %) had been involved in the conduct of RCTs, including using a placebo comparison (60/161 (37 %)), only 49/226 (21 %) of respondents regularly participated in research networks.

**Conclusions:**

Our survey indicated that in the Kingdom of Saudi Arabia (KSA), ICUs are the most likely clinical locations for conducting a clinical trial of convalescent plasma therapy for MERS-CoV, and that most ICUs have experience with such research designs.

**Electronic supplementary material:**

The online version of this article (doi:10.1186/s12871-016-0198-x) contains supplementary material, which is available to authorized users.

## Background

A novel coronavirus was first diagnosed in Saudi Arabia in June 2012 in a man who presented with pneumonia and acute renal failure [1]. This newly discovered virus leading to severe pneumonia and acute respiratory distress syndrome (ARDS) was later named Middle East respiratory syndrome coronavirus (MERS-CoV). As of July 3, 2016, there have been 1769 laboratory-confirmed cases of MERS-CoV infection, 85 % of which occurred in the Kingdom of Saudi Arabia [2]. The case fatality rate is 35 % and reaches 70 % in critically ill patients [3] with no specific therapy available to date.

Convalescent plasma that contains MERS-CoV-specific immunoglobulin and is obtained from previously recovered patients has been suggested as a potential therapy for patients with MERS-CoV infection. Convalescent plasma has been used to treat several other viral infections, including the Severe Acute Respiratory Syndrome (SARS) SARS coronavirus (SARS-CoV), Spanish influenza A (H1N1), avian influenza A (H5N1), and 2009 pandemic influenza A (H1N1 pdm09) [4–10]. A recent meta-analysis of observational studies of passive immunotherapy in SARS and severe influenza suggest reductions in mortality with timely use of convalescent blood products, particularly those with neutralizing antibodies [11]. Public Health England and the International Severe Acute Respiratory & Emerging Infection Consortium (ISARIC) have published a decision-support tool for clinicians managing cases of MERS-CoV infection [12], which relies heavily upon expert opinion and clinical experience of treating patients with severe respiratory disease caused by other viruses including SARS and 2009 pandemic influenza A (H1N1pdm09). The document suggests that current evidence is strongest for testing convalescent plasma (CP) or other therapeutics which contain neutralizing antibodies (such as hyperimmune immunoglobulin) for treatment of serious MERS-CoV illness.

Given the scattered distribution of cases in Saudi Arabia, it is unclear whether the clinical and research infrastructure would be sufficient to support the conduction of a randomized controlled trial (RCT) of convalescent plasma therapy. Such a study will require laboratory ability to diagnose MERS-CoV infection and identify antibody titers among previously infected but recovered patients, research infrastructure and ability to lead or participate in an RCT, and the presence of personal and community equipoise among healthcare workers and patients to enroll in such a trial. Therefore, as part of a collaborative effort among colleagues from the Gulf States and Eastern Mediterranean and with the support of the World Health Organization and International Severe Acute Respiratory and Emerging Infection Consortium, we undertook a survey to assess feasibility of conducting a clinical trial of convalescent plasma therapy for patients with MERS-CoV infection in KSA.

## Methods

### Study population

The study targeted health care specialists in internal medicine, critical care, respiratory disease, infectious disease, hematology, and pediatrics, along with clinical microbiologists, hospital epidemiologists and blood bankers. The survey consisted of voluntary anonymous responses to a web-based questionnaire and did not include actual patient data.

### Questionnaire development

A validated questionnaire, previously used by ISARIC to evaluate and map capacity conduct of clinical trials in response to outbreaks, was used as a basis for the development of this survey. Omitting questions that were deemed not relevant to this study, and modifying or adding questions that were more appropriate to the current objectives led to a questionnaire of 26 items, divided into three main domains of interest. First, four questions aimed to evaluate the ability and capacity of caring for critically ill patients within the KSA health system (e.g., “How many beds are capable of caring for mechanically ventilated patients in the ICU where patients with severe acute respiratory infection (SARI) would usually be treated?”). Second, the study sought to explore laboratory capacity to diagnose MERS-CoV infection and Blood Bank ability to prepare convalescent plasma through seven questions (e.g., “Do you have a mechanism to send specific blood donors (e.g., survivors of MERS-CoV infection) to the blood bank to make donations?” and “What methodologies are available in the laboratories associated with your site for MERS-coV testing?”). The third domain aimed to evaluate research capacity needed to conduct clinical trials and whether or not necessary infrastructure to conduct RCTs existed in KSA, through 10 questions specifically related to research (e.g., “Do you believe that patients at your hospital would participate in randomized studies in which patients are randomly assigned to one of two or more treatments?”). The questionnaire was populated using Survey Monkey, was emailed through the snowball methodology, to physicians who are possibly involved in the care of MERS-CoV patients. Snowball sampling is a chain referral sampling where the initial participants are asked to assist in recruiting more subjects by passing on the questionnaire. We initiated contact with 250 participants from 48 hospitals in Saudi Arabia; those in turn invited others, with a total subjects approached being approximately 582. There were three reminders, 2 weeks apart by email to non-respondents, and the data was collected between March and May 2014. A complete list of the survey questions is included in the online supplement (Additional file 1). The Institutional Review Board (IRB) of Ministry of National Guard Health Affairs (RC14/003/R--29 April 2015) approved this study. The first question of the survey was a question about whether the participant consents to the survey.

### Statistical analysis

Descriptive statistics (frequencies and percentages) were used to quantify the categorical study variables.

## Results

### ICU capacity

Of 582 physicians who were invited to the survey, 327 responded (56.2 %) (Table 1). The professional focus of the majority of respondents was critical care (106/249 (43 %)), pediatrics (59/249 (24 %)) or internal medicine (52/249 (21 %)) but none was blood banking. Nearly all respondents (251/263, 95 %) reported to have access to ICU facilities within their institutions and 44 % of these ICUs had more than 20 beds. Most respondents (219/270 (81 %)) reported that intensivists were the most physician group responsible for treatment decisions about critically ill SARI patients. Available reported therapies in the ICUs included extra-corporal membrane oxygenation (ECMO) (48 %), hemodialysis (91 %) and plasmapheresis (82 %).

**Table 1:** Respondent and ICU characteristics

Questions	Responders to the Question	Answer options	Responders
The professional focus of the respondents^a^	249	Critical care	106 (42.5)
		Pediatrics	59 (23.6)
		Internal medicine	52 (20.9)
		Respiratory	37 (14.8)
		Infectious diseases	34 (13.6)
		Microbiology or virology	7 (2.8)
		Hematology	7 (2.8)
		Blood bank	0
		Other	52 (20.9)
The presence of ICU in the hospital^b^	263	Yes	251 (95.4)
Number of ICU beds capable of caring for mechanically patients ventilated with SARI	285	0–5	37 (12.9)
		6–10	47 (16.4)
		11–20	61 (21.4)
		>20	125 (43.8)
		Unsure	15 (5.2)
Specific therapies available in the ICU	259	Extra-corporeal membrane oxygenation (ECMO)	125 (48.2)
		Hemodialysis	235 (90.7)
		Plasmapheresis and/or plasma exchange	212 (81.8)
		Care to pediatric patients	222 (85.7)
The most responsible physician group for treatment decisions about critically ill patients	270	Intensivists	219 (81.1)
		Pulmonologists	17 (6.2)
		Infectious diseases specialists	17 (6.2)
		Anesthesiologists	9 (3.3)
		Surgeons	1 (0.3)
		Other	7 (2.5)

^a^More than one answer is possible

^b^ICU is defined as a geographic location in the hospital where patients with severe acute respiratory infection (SARI) can be treated with invasive mechanical ventilation

**Table 2:** Laboratory capacity questions

Questions	Responders to the Question	Answer options	Responders	
The source of blood products?	250	Blood bank in your facility	235 (94.0)	
		Blood bank from another facility	15 (6.0)	
Feasibility of obtaining blood donation from specific groups (e.g., survivors of MERS-CoV)	237	Yes	130 (54.9)	
The capability to screen for blood-borne viruses in donated blood (e.g., HIV, hepatitis, etc.)?	248	Yes	240 (96.8)	
Ability to send suspected MERS-CoV samples to a reference laboratory for diagnosis?	226	Yes	173 (76.5)	
Turn-around time	164	1 day	15	
		2 days	24	
		3 days	44	
		4 days	15	
		5 days	24	
		6 days	3	
		7 days	22	
		9 days	1	
		10 days	7	
		14 days	6	
		15 days	1	
		21 days	2	
Methodologies for MERS-CoV testing^a^	205	Real-time PCR	Yes	135
			No	10
			Do not know	57
		Sequencing	Yes	22
			No	21
			Do not know	110
		Serology	Yes	51
			No	21
			Do not know	88
		Other (please specify)		8
Available diagnostic work-up for severe acute respiratory infection^a^	218	Blood cultures	Yes	205
			No	4
			Do not know	6
		Urine bacterial antigen testing	Yes	153
			No	28
			Do not know	30
		Viral antigen testing	Yes	146
			No	36
			Do not know	28
		PCR for bacterial and viral testing	Yes	170

^a^More than one answer is possible

**Table 3:** Research capacity questions

Questions	Responders	Answer options	Responders
Regular participation of the respondent in a specific research network(s)?	226	Yes	49 (21.7)
The predominant focus of the network^a^	114	Clinical infectious diseases	46 (40.3)
		Microbiology or virology	24 (21.0)
		Critical care	71 (62.2)
		Public or global health	34 (29.8)
		Other (please specify)	11 (9.6)
Participation in research initiatives^a^	148	Local clinical research initiatives	130 (87.8)
		National clinical research initiatives	67 (45.3)
		International clinical research initiatives	55 (37.2)
Age categories of patients are recruited into studies at the respondent site^a^	165	Adult	139 (84.2)
		Pediatrics	56 (33.9)
Settings in the hospital that conduct clinical research^a^	165	Emergency department	66 (40.0)
		Hospital wards	104 (63.0)
		ICUs	125 (75.8)
		Outpatient/community care settings	75 (45.5)
		Other	5 (3.0)
Research types in the respondent hospital^a^	161	Retrospective observational studies^b^	147 (91.3)
		Prospective observational studies	115 (71.4)
		Biological sampling studies (blood and other fluids)	79 (49.1)
		Randomized clinical trials of interventions	80 (49.7)
		Clinical trials using a placebo control comparison group	60 (37.3)
Do you believe that patients at your hospital would participate in randomized studies?	181	Definitely not	8 (4.4)
		Probably not	31 (17.1)
		Maybe	70 (38.7)
		Probably yes	49 (27.1)
		Definitely	23 (12.7)
The presence of registry data (i.e., an electronic or hard-copy database)	179	Yes	146 (81.6)

^a^More than one answer is possible

^b^Retrospective observational studies include case reports, chart review case series, case-control studies, cohort studies

### Laboratory capacity

Majority of the respondents (235/250 (94 %)) indicated that blood products used in their hospitals were obtained in the same hospital, only 15/250 (6 %) reported that blood products are obtained from a blood bank at another institution. When asked about the feasibility of having MERS-CoV infection survivors to donate blood/plasma, 130/237 (55 %) of the respondents reported this would be feasible (Table 2). A majority (240/248 (97 %)) of the respondents reported to have access to the necessary facilities and capabilities for screening for blood-borne viruses in donated blood within their institutions. In regards to laboratory testing for MERS-CoV, 173/226 (77 %) reported that samples for MERS-CoV testing were sent to reference laboratories for diagnosis, with 147/164 (90 %) reported that the results were obtained within seven days. Other microbiologic tests for pneumonia work-up were reported to be available as follows: 95 % blood cultures, 73 % for urine bacterial antigen testing, 70 % for viral antigen testing, and 79 % for PCR for bacterial and viral testing. Respondents reported that the most commonly available diagnostic test was real-time PCR (67 %) and the least available methodology was gene sequencing (14 %).

### Research capacity

Most respondents reported involvement in local clinical research initiatives (88 %) while only 21 % of respondents claimed to be part of specific research networks, predominantly focused upon clinical infectious diseases and critical care and the focus of investigation was mostly (84 %) upon adults. Most research (76 %) is perceived to take place in ICUs (76 %) and hospital wards (63 %) (Table 3). Of the physicians surveyed, 91 % worked at hospitals that have participated in retrospective observational studies, while 71 % reported being involved in prospective studies or trials. Only half of the respondents (49 %) perceived their institutions had been involved with biological sampling studies (of blood and other fluids) or randomized clinical trials of interventions (50 %), while only 37 % knew of their institutions having been involved in clinical trials using a placebo control comparison group.

A majority 146/179 (82 %) of respondents said that they had access to registry data for patients receiving care at their sites, and more than 50 % of respondents reported that their institutions had a research administration infrastructure in place to aid with data-sharing agreements (52 %), research contract (49 %), ethics reviews (74 %), and administrative support for research projects (60 %). The average time for the ethical review process (from submission to approval to enroll patients) was approximately 1 to 3 months.

Although 22 % of respondents said that the patients at their hospital were definitely not or probably not likely to participate in randomized studies and 39 % said that they were ‘maybe’ prepared to parttake in such studies, 40 % of respondents said they believed their patients would probably or definitely agree to participate.

## Discussion

Our survey identifies that many healthcare facilities in KSA have the necessary capacity to participate RCTs that enroll severely ill patients with MERS-CoV infection. The survey highlights a few important points in support of this conclusion. First, most respondents believed that the needed infrastructure was in place – such as data registries, laboratory capacity, ethics review processes, and monitoring units for RCTs. Second, respondents seemed to be aware of the required capacity for research in MERS-CoV. Third, although not the most common trial design in Saudi Arabia, most respondents were aware of RCTs and what they entail, and a majority of them believed that their patients would consider participating in RCTs. However, many barriers to conducting RCTs remain. First, there is low community awareness of research in general, which is needed for any RCT to take place. Second there may be lengthy processes required to obtain regulatory and ethics approval to initiate clinical trials.

This survey suggests that the necessary clinical and laboratory facilities exist to conduct such RCTs. The presence of ICUs with sufficient numbers of beds is a necessary component of conducting RCTs on potential therapies for critically ill patients with MERS-CoV. Essential laboratory infrastructure for such studies are also available including blood banking, microbiologic diagnostic testing for pneumonia in general and MERS-CoV in particular. However, as none of the respondents was a blood bank specialist, it is not possible to explore this in greater detail on the basis of this survey alone. A majority of the respondents (54.9 %) reported that sending individuals who had recovered from MERS-CoV to donate blood is feasible, which is a positive and necessary step for obtaining convalescent plasma.

There are a number of limitations associated with this study. Because of the snowball methodology, the number of subjects who were approached is only approximate. Response rate was modest, and response rates to individual questions varied. Due to the anonymity of replies to this survey it has not been possible to follow up on incomplete responses. There were no responses from blood banks. As with other surveys, it is possible that individuals motivated to conduct research or work in institutions that conduct research were more likely to respond to the survey; creating a bias towards more favorable answers for research infrastructure and culture. The study did not include a face-to-face interview, which would have resulted in higher targeted responses. Finally, surveys represent stated responses as opposed to measurement of actual practice.

## Conclusions

Our survey results indicate that the research infrastructure at many acute care facilities in Saudi Arabia is likely generally sufficient to conduct a RCT to investigate the efficacy of convalescent plasma treatment for severely ill patients with MERS-CoV. Next steps in such a research program will need to include an observational study of actual clinical and diagnostic practice in the care of patients with MERS-CoV in KSA. This would help to inform design of a pilot clinical trial with a goal to demonstrate feasibility and safety of convalescent plasma evaluation, and would need to occur before a true evaluation of efficacy could take place.

## Abbreviations

CP, convalescent plasma; ECMO, extra corporal membrane oxygenation; HCW, health care workers; ICU, intensive care unit; ISARIC, International Severe Acute Respiratory & Emerging Infection Consortium; KSA, Kingdom of Saudi Arabia; MERS-CoV, Middle East respiratory syndrome coronavirus; PCR, polymerase chain reaction; RCT, randomized controlled trial; SARI, severe acute respiratory infection; SARS, severe acute respiratory syndrome; WHO, World Health Organization

## Additional file

Additional file 1: Severe Acute Respiratory Infection Survey for Regions affected by Middle Eastern Respiratory Syndrome Coronavirus (MERS-CoV) (DOC 238 kb)
