# Supplementary material for: Feasibility of a randomized controlled trial to assess treatment of Middle East Respiratory Syndrome Coronavirus (MERS-CoV) infection in Saudi Arabia: a survey of physicians
Source: BMC Anesthesiol. 2016 Jul 12;16:36. doi: 10.1186/s12871-016-0198-x (PMC4942900; doi:10.1186/s12871-016-0198-x)
Supplement: Additional file 1: — Severe Acute Respiratory Infection Survey for Regions affected by Middle Eastern Respiratory Syndrome Coronavirus (MERS-CoV) (DOC 238 kb) [file 12871_2016_198_MOESM1_ESM.doc]

# Severe Acute Respiratory Infection Survey for Regions affected by Middle Eastern Respiratory Syndrome Coronavirus (MERS-CoV)

**Introduction and participation**

Dear Colleague,

We would like to invite you to the following survey. Please fill this survey if your specialty if you are one of the following clinicians:

• Internal Medicine Clinicians
• Critical Care Clinicians
• Respiratory Diseases Clinicians
• Infectious Diseases Clinicians
• Clinical Microbiologists
• Hospital Epidemiologists
• Blood Bank and Haematology Clinicians

Pediatric Clinicians

This survey is a collaboration among colleagues from the Gulf States and Eastern Mediterranean regions who have been involved in caring for patients with, or investigating the outbreak of, MERS-CoV, with support from the World Health Organization (WHO) and International Severe Acute Respiratory and Emerging Infection Consortium (ISARIC). The data gathered from this survey will help inform what clinical research could be readily initiated to further study MERS-CoV illness and its management.

One potential therapy that has been identified for evaluation among patients infected with MERS-CoV is the use of convalescent plasma from previously recovered patients, or MERS-CoV-specific immune globulin.

Thank you very much for considering to participate.


Sincerely,

**The Severe Acute Respiratory Infection Survey**

1. If you agree to participate please confirm below

| Yes  No |
| --- |

**Identification - of the person filling in the survey (optional)**

**2. What is your name? (Optional)**

**3. What is your email address? (Optional)**

**4. W**hat is your professional focus? (Please select all that apply)

| What would you say is your professional focus? (Please select all that apply)  Internal medicine  Infectious diseases  Microbiology or virology  Critical care  Respiratory  Hematology  Blood banking  Pediatrics |
| --- |
| Other (please specify) |

**Level of care available - at your site**

**5**. Does your site have an intensive care unit? (i.e. a geographic location in the hospital where patients with Severe Acute Respiratory Infection - SARI - can be treated with invasive mechanical ventilation)

| Yes  No |
| --- |

**6. How many beds are capable of caring for mechanically ventilated patients in the ICU where patients with Severe Acute Respiratory Infection (SARI)** would usually be treated? (select or complete one)

| 0-5  6-10  11-20  >20  Unsure |
| --- |
| Approximate number: |

**7**. Can ICUs in your hospital provide the following types of care?

|  | **YES** | **NO** |
| --- | --- | --- |
| **Extra-corporeal Membrane Oxygenation (ECMO)** |  |  |
| **Hemodialysis** |  |  |
| **Plasmapheresis and/or Plasma Exchange** |  |  |
| **Care to pediatric patients** |  |  |

|  |
| --- |

**8. Which is 'the most responsible physician group' primarily in charge of making treatment decisions about critically ill Severe Acute Respiratory Infection (SARI)** patients in this ICU?

| Intensive care specialists  Anesthesiologists  Infectious diseases specialists  Surgeons  Pulmonologists |
| --- |
| Other (please specify) |

**Issues related to Convalescent plasma**

| **9. What is the source of blood products for your patients?**  Blood bank in your facility  Blood bank from another facility |
| --- |

**10**. Do you have a mechanism to send specific blood donors (e.g. survivors of MERS-CoV) to the blood bank for making donations?

| Yes  No |
| --- |

**11** Does your site or other facility have the capability to screen for blood borne viruses in donated blood (e.g. HIV, Hepatitis, etc.)?

| Yes  No |
| --- |

**Laboratory capability at your site**

**12. Do you send suspected MERS-CoV samples out to a reference laboratory for diagnosis? (If your answer is 'No', please proceed to Q14**)

| Yes  No |
| --- |

**13. If you answered 'Yes' to Q12, please indicate how long it takes to receive results below, and proceed to Q16.**

**14. What methodologies are available in the laboratories associated with your site for MERS-CoV testing? (Please select all that apply)**

|  | **Yes** | **No** | **Do not know** |
| --- | --- | --- | --- |
| **Real-time (RT)-PCR** |  |  |  |
| **Sequencing** |  |  |  |
| **Serology** |  |  |  |

Other (please specify)

**15. For patients with severe acute respiratory infection, does you facility have the ability to provide the following diagnostic testing: (Please select all that apply)**

|  | **Yes** | **No** | **Do not know** |
| --- | --- | --- | --- |
| **Blood cultures** |  |  |  |
| **Urine bacterial antigen testing** |  |  |  |
| **Viral antigen testing** |  |  |  |
| **PCR for bacterial and viral testing** |  |  |  |

**Research activity and infrastructure at your site**

**16**. Do you regularly participate in a specific research network(s)?

| Yes  No |
| --- |

**17. If you answered 'Yes' to Q16, what is/are the name(s) of the research network?**

**18**. What is the predominate focus of the network? (Please select all that apply)

| Clinical infectious diseases  Microbiology or virology  Critical care  Public or global health |
| --- |
| Other (please specify) |

**Location of activity and collaborators**

**19**. Do you participate in: (Please select all that apply)

| Local clinical research initiatives  National clinical research initiatives  International clinical research initiatives |
| --- |

**20**. What age categories of patients are recruited into clinical research studies run by your site? (Please select all that apply)

| Adult  Pediatric |
| --- |

**21**. In what settings does your hospital conduct clinical research, as far as you know? (Please select all that apply)

| Emergency department  Hospital wards  ICUs  Out-patient/Community care settings |
| --- |
| Other (please specify) |

**22**. Does your hospital participate in the following kinds of research: (Please select all that apply)

| Retrospective observational studies (case reports, chart review case series, case-control studies, cohort studies)  Prospective observational studies  Biological sampling studies (blood and other fluids)  Randomized clinical trials of interventions  Clinical trials using a PLACEBO control comparison group |
| --- |

**23. Do you believe that PATIENTS at your hospital, would participate in randomized studies (where patients are randomly assigned to one of two or more treatments):**

|  | **Definitely not** | **Probably not** | **Maybe** | **Probably yes** | **Definitely** |
| --- | --- | --- | --- | --- | --- |
|  |  |  |  |  |  |

**24**. Does your site collect or have access to registry data from your patients? (i.e. an electronic or hard-copy database of patients receiving care at your site)

| Yes  No |
| --- |

**25**. Does your institution have a research administration infrastructure to aid with: (Please select all that apply)

| Data-sharing agreements  Research contract review  Research ethics review  Research project administrative support |
| --- |

**26**. What is the process of obtaining review and approval for research at your site? (Please select all that apply)

| Submission to research ethics board/committee  Submission to hospital administration  Submission to a department or division head  Submission to local government health official  Submission to other group (please specify below)  It depends on the type of study or trial |
| --- |
| Other (please specify) |

**27**. For what type of study would you have to seek research ethics approval? (Please select all that apply)

| Retrospective observational study (e.g. case review)  Prospective observational study (collecting data on new patients with a condition of interest)  Biological sampling study (collecting fluids or other samples from patients)  Clinical trial (comparing an experimental treatment to a placebo or usual best care) |
| --- |

**28**. How long does the process normally take from time of submission of a completed proposal to research ethics approval to enroll patients?

| 1 week  1 month  2 months  3 months  Longer |
| --- |
